# Does Gerofit Exercise Reduce Veterans’ Use of Emergency Department and Inpatient Care?

**DOI:** 10.1093/geroni/igaa057.2786

**Published:** 2020-12-16

**Authors:** Lauren Abbate, Jiejin Li, Peter Veazie, Orna Intrator, Cathy Lee, Leslie Katzel, Megan Pearson, Miriam Morey

**Affiliations:** 1 Rocky Mountain Regional VA Medical Center, Aurora, Colorado, United States; 2 Geriatrics and Extended Care Data and Analysis Center (GECDAC), Canandaigua, New York, United States; 3 University of Rochester School of Medicine and Dentistry, Rochester, New York, United States; 4 VA Greater Los Angeles, Los Angeles, California, United States; 5 Geriatric Research, Education, and Clinical Center, VA Maryland Health Care System, Baltimore, Maryland, United States; 6 Veteran Affairs Health Care System, Durham, North Carolina, United States; 7 Veterans Health Administration, Durham, North Carolina, United States

## Abstract

Little is known about the relationship between exercise and health care utilization in older adults. This study examined hospitalizations/emergency Department (ED) visits in the 12 months prior to and during 12 months of active Gerofit participation (across 5 sites). Data were compared for each outcome to a propensity matched nearest neighbor sample from the same site [Mean, 95% CI]. Of the 226 Veterans who were active in the program for ≥12 months and enrolled in VA and Traditional Medicare for 12 months prior to Gerofit participation, hospitalizations/ED visits were greater prior to (15.3%/42.0%) than during (6.8%/37.1%) Gerofit participation. Gerofit participants were 8% less likely to have a hospitalization in the 12 months following enrollment than controls [-0.08 (-0.14, -0.02)] but no between-group differences in ED use [-0.00 (-0.11, 0.10)] were observed. Participation in Gerofit may reduce hospitalizations, but its impact on ED use is inconclusive.

